# Magnetic targeting enhances retrograde cell retention in a rat model of myocardial infarction

**DOI:** 10.1186/scrt360

**Published:** 2013-12-12

**Authors:** Zheyong Huang, Yunli Shen, Aijun Sun, Gangyong Huang, Hongmin Zhu, Bingqing Huang, Jianfeng Xu, Yanan Song, Ning Pei, Jing Ma, Xiangdong Yang, Yunzeng Zou, Juying Qian, Junbo Ge

**Affiliations:** 1 Shanghai Institute of Cardiovascular Diseases, Zhongshan Hospital, Fudan University, 180 Feng Lin Road, Shanghai 200032, China; 2 Department of Cardiology, Shanghai East Hospital, Tongji University, 150 Jimo Road, Shanghai 200120, China; 3 Department of Orthopedics, Huashan Hospital, Fudan University, 12 Middle Urumqi Road, Shanghai 200040, China; 4 College of Science, Shanghai University, 99 Shangda Road, Shanghai 200444, China; 5 Department of Radiology, Zhongshan Hospital, Fudan University, 180 Feng Lin Road, Shanghai 200032, China; 6 Institute of Biomedical Science, Fudan University, 180 Feng Lin Road, Shanghai 200032, China

## Abstract

**Introduction:**

Retrograde coronary venous infusion is a promising delivery method for cellular cardiomyoplasty. Poor cell retention is the major obstacle to the establishment of this method as the preferred route for cell delivery. Here, we explored whether magnetic targeting could enhance retrograde cell retention in a rat model of myocardial infarction.

**Methods:**

Rat mesenchymal stem cells were labeled with superparamagnetic oxide nanoparticles. The magnetic responsiveness of MSCs was observed while cells flowed through a tube that served as a model of blood vessels in a 0.6-Tesla magnetic field. In a Sprague–Dawley rat model of acute myocardial infarction, 1 × 10^6^ magnetic mesenchymal stem cells were transjugularly injected into the left cardiac vein while a 0.6-Tesla magnet was placed above the heart. The cardiac retention of transplanted cells was assessed by using quantitative Y chromosome-specific polymerase chain reaction, cardiac magnetic resonance imaging, and optical imaging. Cardiac function was measured by using echocardiography, and histologic analyses of infarct morphology and angiogenesis were obtained.

**Results:**

The flowing iron oxide-labeled mesenchymal stem cells were effectively attracted to the area where the magnet was positioned. Twenty-four hours after cellular retrocoronary delivery, magnetic targeting significantly increased the cardiac retention of transplanted cells by 2.73- to 2.87-fold. Histologic analyses showed that more transplanted cells were distributed in the anterior wall of the left ventricle. The enhanced cell engraftment persisted for at least 3 weeks, at which time, left ventricular remodeling was attenuated, and cardiac function benefit was improved.

**Conclusions:**

These results suggest that magnetic targeting offers new perspectives for retrograde coronary venous delivery to enhance cell retention and subsequent functional benefit in heart diseases.

## Introduction

Cell therapy is a promising approach for acute myocardial infarction (AMI) and heart failure, and its efficacy largely depends on cell homing, retention, and engraftment within the injured myocardium. With unique access to the ischemic myocardium, retrograde coronary venous delivery has been demonstrated to provide efficient cell dissemination in the setting of occluded or diffusely narrowed coronary arteries and has subsequently shown functional benefits in both animal and clinical studies [1-6]. However, compared with the antegrade approach, cell retention using the retrograde intracoronary approach was inferior [7-9]. Poor cell retention is the major obstacle in establishing this method as the preferred route for cell delivery.

In recent years, magnetic targeting strategies traditionally used in chemotherapy for tumors [10] have been introduced to localize magnetic nanoparticle-loaded cells delivered to *in vivo* target lesions [11-14]. Until now, magnetic targeting strategies have been successfully introduced to attract cells infused via intramyocardial [15] and intracoronary [16,17] routes to the ischemic heart. This technique has been proven to enhance cell retention, engraftment, and functional benefits. However, few data exist on the efficacy of magnetic targeting on retrograde cell retention.

Based on a new transjugular cardiac vein retroinfusion technique [18] and an analysis of the interaction between a magnet cylinder and the magnetically labeled MSCs, here we explored whether magnetically targeted cell delivery could enhance myocardial retention of MSCs after retrograde coronary vein infusion in a rat model of myocardial infarction.

## Methods and materials

### Magnet cylinder

A permanent neodymium-iron-boron (NdFeB) magnet cylinder with a diameter of 8 mm (Shanghai Yahao Instrument Equipment Co., China) was used in this study. The magnetic flux density (B) of the magnet surface was up to 600 mT, measured by using a model 51,662 Leybold Tesla meter. The distribution of the magnetic flux density was calculated with finite element analysis.

### Preparation of magnetically labeled cells

Bone marrow MSCs were isolated from 4-week-old male Sprague–Dawley (SD) rats weighing 100 to 120 g, as described before [19,20]. All cells used for the subsequent experiments were harvested with 0.25% trypsin when they reached 80% to 90% confluence at passage 4.

MSCs were labeled with superparamagnetic iron oxide nanoparticles (SPIO; Schering, Berlin, Germany; 100 mg/ml, 62 nm in diameter) and poly-L-lysine (PLL; 0.15 mg/ml), with an iron concentration of 50 μg/ml and a PLL concentration of 0.15 μg/ml [19]. The magnetic SPIO-labeled MSCs (MagMSCs) were then incubated with 1 μ*M* ethyl iodotricarbocyanine iodide (DiR; ABD Bioquest, Inc., California, USA) according to the manufacturer’s protocol. Prussian blue staining and transmission electron microscopy (Philips CM120) were used to evaluate the presence and localization of intracellular iron particles. The iron content in the cells was quantified by using atomic absorption spectrometry (Thermo E.IRIS Duo ICP). Inverted microscopy was used to examine the staining efficacy of the DiR dye.

### Proliferation assays and determination of viability

For the proliferation and viability assays, the following conditions were investigated: unlabeled MSCs without exposure to magnetic fields (MSCs), unlabeled MSCs with exposure to magnetic fields (Mag-MSCs), SPIO-labeled MSCs without exposure to magnetic fields (SPIO-MSCs), and SPIO-labeled MSCs with exposure to magnetic fields (Mag-SPIO-MSCs). For the exposure to magnetic fields, the 75-cm^2^ cultures were positioned above and in direct contact with the magnets for 24 hours.

In the proliferation assays, cells were seeded at 3 × 10^5^ cells/flask, and the medium was changed every 3 days. At subconfluence (90%), the cells were detached with Accutase (PAA Laboratories, Cölbe, Germany) and counted with a CASY2 Analyser (CASY2-Cell Counter and Analyser System, Model TT, Roche Diagnostics, Mannheim, Germany) [21]. The experiments were performed in triplicate. Cellular viability under the different conditions was examined with the help of a CASY2 Analyser, according to the ECE method described by Lindl *et al*. [22] as well as the viability-SOP of the manufacturer.

### *In vitro* magnetic capture of flowing MagMSCs

To test the magnetic responsiveness of MagMSCs, the accumulation of MagMSCs was observed while cells flowed through a tube, serving as a model of blood vessels in a magnetic field. In total, 20 ml MagMSC suspension at a concentration of 5 × 10^4^ cells/ml was placed in a 50-ml syringe and flowed through a quartz tube (ID 2.3 mm, OD 4.3 mm, length 20 mm). The aforementioned magnet cylinder was placed tightly at the midsegment of the tube. The cell suspension flowed through the magnet field and was collected in centrifuge tube 1. The remaining cells within the quartz tube were washed by using DMEM–LG culture medium and collected in centrifuge tube 2. The numbers of MagMSCs in centrifuge tubes 1 and 2 were counted by using a counting plate and referred to as either the quantity of captured cells (Q_1_) or the quantity of uncaptured cells (Q_2_). The capture efficiency (CE) was calculated by

$$\mathrm{CE}=\frac{Q_{1}}{Q_{1}+Q_{2}}\times 100\%$$

The cell suspension circulated at flow rates of 4 mm/s, 20 mm/s, 100 mm/s, and 500 mm/s to mimic circulatory conditions in animals and human beings. Then the effects of the fluid velocity on the accumulation of MagMSCs were observed. All experiments were performed in triplicate for each condition.

### Acute myocardial infarction model

The animal experiments were approved by the Animal Care and Use Committee of Fudan University and were in compliance with the *Guide for the Care and Use of Laboratory Animals*, published by the National Academy Press (NIH Publication No. 85–23, revised 1996). An experimental anterior-wall MI was induced, as previously described by Antonio *et al.*[23]. In brief, the rats were anesthetized with ketamine (100 mg/kg, i.p.) and xylazine (10 mg/kg, i.p.), and a rodent ventilator was used to ventilate rats with room air at 80 breaths per minute. The heart was exteriorized through a left thoracotomy, and the left anterior descending coronary artery (LAD) was occluded with a 6–0 polypropylene suture.

### Transjugular cardiac vein catheterization and its perfusion area

Half an hour after LAD ligation, a transjugular cardiac vein catheterization was induced under additional anesthesia with ketamine (30 mg/kg, i.p.), as we reported recently [18]. In brief, a flexible wire with a bent end was inserted into the left internal jugular vein and advanced slowly along the left superior vena cava. Under direct vision, the wire was run into the left cardiac vein by rotating the wire and changing the position of its tip. A fine tube was advanced along the wire to the proximal-mid segment of the left cardiac vein. A temporal snare suture was placed at the distal left cardiac vein to prevent flush back of injected solution into the left vena cava.

To examine the perfusion area of the transjugular cardiac vein catheter, 1 ml of 0.4% Evans blue solution was injected slowly and selectively into the left cardiac vein while the snare was tied. Five minutes later, the snare was released to allow reperfusion, and the catheter was removed. The heart was removed after 5 minutes of observation and cut into five segments parallel to the apex-base axis. The perfusion area of the retrograde infusion was compared with the ischemic area induced by LAD ligation. The latter was evaluated with triphenyltetrazolium chloride (TTC) staining.

### *In vivo* magnetic targeting of retrograde delivered MagMSCs

**Figure 1:**
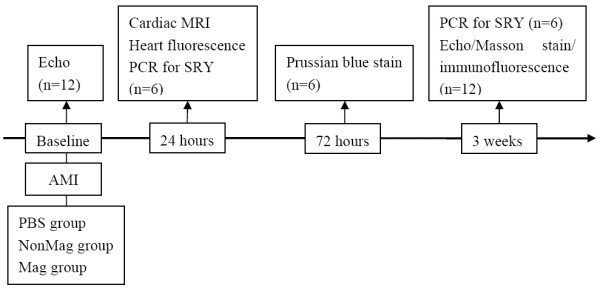
**Study protocol.** Echo, echocardiography; AMI, acute myocardial infarction; MRI, magnetic resonance imaging; SRY, sex-determining region Y gene.

Figure 1 summarizes the diagnostic and surgical steps of the animal experiments. A rat AMI model was developed in female Sprague–Dawley (SD) rats (150 to 200 g). In total, 90 surviving rats were randomly divided into three treatment groups. The Mag group received 1 × 10^6^ Mag-Dir-MSCs with magnetic guidance (*n* = 30). The NonMag group received 1 × 10^6^ Mag-Dir-MSCs without magnetic guidance (*n* = 30), and the PBS group received PBS alone (*n* = 30).

In the Mag group, 20 minutes after the establishment of transjugular cardiac vein access in the AMI model, 1 × 10^6^ Mag-Dir-MSCs were resuspended in 1 ml PBS and injected slowly and selectively into the left cardiac vein while the snare was tied. Five minutes later, the snare was released to allow reperfusion, and the catheter was removed. For magnetic targeting, the cylindrical NdFeB magnet was placed close to the injured myocardium (0 to 1 mm) for 10 minutes during and after the cell injection, which has been suggested to be the optimized time [15,17]. The surgical wounds were repaired, and the rats were extubated and returned to their cages to recover.

### Cardiac magnetic resonance imaging at 24 hours after injection

To determine the efficacy of magnetic targeting, six animals from each group were killed for magnetic resonance imaging (MRI) at 24 hours after the injection of cells or PBS. MRI was performed in a 3.0-T clinical whole-body magnetic resonance scanner by using a 5-cm-diameter four-element phased-array animal coil (MAGNETOM Verio; Siemens Healthcare). Images were acquired by T_2_ fl2d sequence with a slice thickness of 1.5 mm, a flip angle of 20 degrees, a field of view of 80 mm, a bandwidth of 240, a matrix of 256 × 256, a repetition time of 150 ms, and an echo time of 4.69 ms. After *in vivo* imaging, the animals were killed. The hearts were explanted for fluorescence examination and then placed in liquid nitrogen and stored at −80°C for further RT-PCR analysis.

Consecutive short-axis slices were acquired to analyze the signal intensity in the myocardium of the left ventricle with Image J software (NIH, Bethesda, MD, USA). The relative signal intensity of the anterior wall was calculated as the signal intensity in the anterior wall divided by the signal intensity in the interventricular septum.

### Cardiac fluorescence imaging at 24 hours after injection

After cardiac MRI, the hearts of six animals were explanted for fluorescence imaging. Extensive PBS washing was performed to remove any cells adhering to the epicardium. The hearts were placed in a Carestream In-Vivo Multispectral Imaging System FX PRO to detect DiR fluorescence under 748-nm excitation and 780-nm emission. The exposure time was set at 3 seconds and was maintained during the entire imaging session. Hearts from the PBS group were also imaged as controls for background noise.

### Quantification of male-donor MSCs in rat heart

As a complementary means of measuring cell retention, we transplanted male MSCs into female recipient hearts to enable the use of Y chromosome-specific PCR to quantify the exact numbers of cells per milligram of heart tissue [24]. Quantitative PCR for the Sex-determining Region Y gene (*SRY*) was performed 24 hours (after fluorescence imaging) and 3 weeks after cell infusion. The primers for specific rat Y chromosome DNA are as follows: sense: 5'-GAG CTT TGG GAG CAG TGA C-3', anti-sense: 5'-ATG AGG CTG ATA TTT ATA GTT TGG-3'. Cell retention was calculated as the total number of cells detected in the heart divided by the number of infused cells.

### Assessment of cardiac function

Cardiac remodeling and left ventricular function were assessed by transthoracic echocardiography 3 weeks after AMI by using a Vevo 770 high-resolution imaging system (Visual Sonics) with a 17.5-MHz probe. After the induction of light general anesthesia, the hearts were imaged in the two-dimensional and M-modes. The recordings were obtained from the parasternal long-axis view at the level of the greatest LV diameter. The LV internal end-diastolic diameter (LVEDd) and LV internal end-systolic diameter (LVEDs) were measured from M-mode recordings, according to the leading-edge method. All echocardiographic measurements were averaged from at least five separate cardiac cycles. All procedures and analyses were performed by an experienced researcher who was blinded to the groups.

### Histologic analysis

Because vascular delivered cells reportedly translocate into the parenchyma after 48 to 72 hours [25], the distribution of infused cells within the hearts was analyzed at 72 hours after injection of cells or PBS. Six hearts from each group were stained with Prussian blue, and another six hearts from each group were frozen in OCT for cell fluorescence imaging. For Prussian blue staining, pathological specimens were incubated for 30 minutes with 2% potassium ferrocyanide (Perl reagent) in 6% hydrochloric acid and were counterstained with nuclear fast red.

The representative hearts were harvested and frozen in OCT compound 3 weeks after rat echocardiography. Cryostat sections (8 μm thickness) were obtained for immunofluorescence staining to evaluate cardiomyocyte differentiation of injected MSCs. Antibodies used included troponin T (TnT, cardiac isoform Ab-1; Laboratory Vision Corp., Chicago, IL, USA), as described previously [26]. Capillary density was assessed by staining with anti-CD31 antibody (BD Biosciences, San Diego, CA, USA), as previously described [27]. The sections were visualized by fluorescence-labeled secondary antibodies (Molecular Probes, Inc., Eugene, OR, USA). In total, 10 visual fields where a cross section of capillaries was clearly visible in the peri-infarct zone were randomly selected, and the numbers of capillaries per square millimeter were obtained after superimposing a calibrated morphometric grid on each digital image by using ImageJ Software.

For morphometric analysis, six representative recipient hearts in each group were harvested 3 weeks after functional assessment and were embedded in paraffin. From the Masson’s trichrome-stained images, morphometric parameters including risk area and the LV wall thicknesses in the risk and noninfarcted regions were measured in each section [28]. Images were acquired digitally and analyzed by using the NIH ImageJ software. All analyses were performed in a blinded manner.

### Statistical analysis

The data are expressed as the mean ± SD. The numbers of transplanted cells from the Mag and the NonMag groups were compared by using Student *t* test. The data from the three groups were compared, and intergroup differences were analyzed with one-way ANOVA (analysis of variance) and the least-significant difference (LSD) test. The relation between the accumulation percentage of MagMSCs and the fluid average velocity was analyzed by the Pearson correlation test. Statistical analyses were performed with SPSS 15.0. A value of *P* < 0.05 was considered statistically significant.

## Results

### Magnetic analysis of the NdFeB magnet cylinder

**Figure 2:**
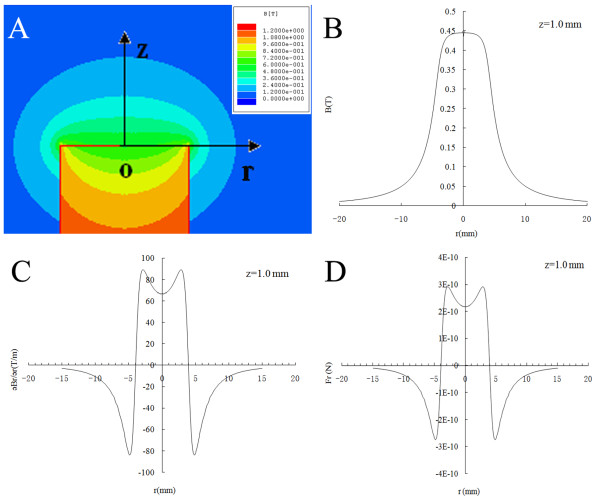
**The magnetic field of the *Nd*FeB magnet cylinder*. *(A)** The system of coordinates and flux-density plot of the NdFeB magnet cylinder*. O* denotes the center point of the surface of the magnet pole. *r* and *z* denote the horizontal and vertical distance from the *O* point, respectively. **(B)** The distribution of the magnetic flux density. **(C)** The distribution of the magnetic flux gradient. **(D)** The magnetic attraction force on MagMSCs in the *r* direction (*Fr*) when *z* is 1 mm. *B*, Magnetic flux density; ($\frac{\partial B}{\partial r}$); Magnetic field gradient in the *r* direction; *Fr*, magnetic attraction force on MagMSCs.

The distribution of magnetic flux density created by the NdFeB permanent magnet cylinder was calculated by finite-element analysis (Figure 2A). The magnetic density decreased as the distance from the surface of the magnet increased and increased as the distance from the center of the magnet increased, reaching its maximum at its flank. Because the working magnetic site was at approximately a 1-mm distance from the surface of the magnet (*z* = 1 mm) in this experiment, we therefore analyzed the distributions of the magnetic flux density, the magnetic flux gradient, and the magnetic attraction force on MagMSCs in the *r* direction (*Fr*) when *z* is 1.0 mm (Figure 2B-D). The *Fr* was theoretically based on measures such as the iron content, SPIO thickness, cell size, and the distribution of the magnetic flux density, as we described previously [19].

### Characterization of MagMSCs

**Figure 3:**
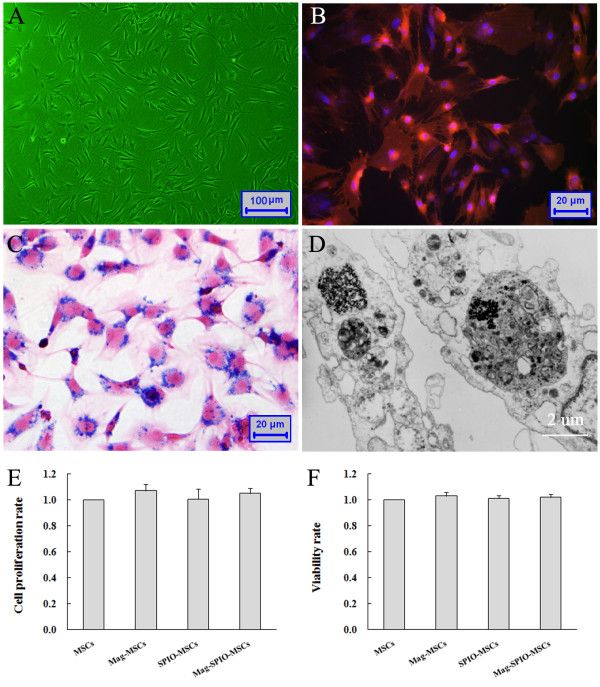
**Cell preparation. (A)** Unlabeled fourth-passage MSCs in culture. **(B)** Fluorescence microscopy images of MSCs labeled with magnetic nanoparticles and DiR stain (Mag-Dir-MSCs) expressing strong red fluorescence. Blue indicates the DAPI nuclear stain. **(C)** Prussian blue staining of Mag-Dir-MSCs on glass coverslips. The magnified image shows blue magnetic particles distributed around cell nuclei as a spherical shell. **(D)** Transmission electron microscopy of Mag-Dir-MSCs. The black compact particles are magnetic nanoparticles that were successfully internalized into the cells. **(E, F)** Proliferation rate and viability rate of MSCs. No significant differences were detected among unlabeled MSCs and labeled MSCs with and without exposure to magnetic fields.

The Mag-Dir-MSCs had a strong red fluorescence signal (Figure 3B). The Prussian blue staining of the MSCs showed intracytoplasmic iron inclusions as dense blue-stained vesicles, and the magnetic nanoparticles were distributed evenly around the nucleus of the MSCs as a spherical shell (Figure 3C). The labeling efficiency was approximately 100% reproducible by using a very low concentration of iron oxide (50 μg/ml). Transmission electron microscopy of labeled cells indicated the presence of anionic magnetic nanoparticles exclusively in polydisperse vesicles in the cytoplasm, and no obvious change in the ultramicrostructure was observed (Figure 3D). The average size of the magnetic vesicles was approximately 500 nm. The average cellular iron content was 21.77 ± 3.62 pg per cell after 24 hours of incubation at 37°C, with 50 μg/ml SPIO in the medium. The cell size ranged between 6 μm and 36 μm (average, 19.3 μm) in suspension. The proliferation rate and viability rate showed no differences between unlabeled MSCs and labeled MSCs with and without exposure to magnetic fields (Figure 3E,F).

### *In vitro* magnetic capture of flowing MagMSCs

**Figure 4:**
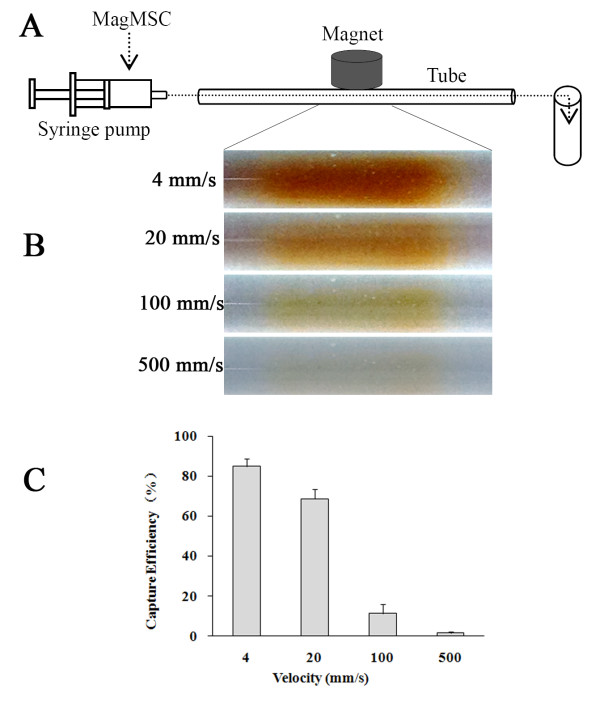
**Magnetic concentration of the flowing MagMSCs. (A)** A schematic illustration of the microfluidic system. The MagMSCs were injected into the tube, and the flow rate was controlled by using a syringe pump. Cells were captured by using an NdFeB magnet located beside the tube. **(B)** Representative images of cell accumulation in the segment close to the magnet at velocities of 4 mm/s, 20 mm/s, 100 mm/s, and 500 mm/s. **(C)** The efficiency of magnetically captured flowing MagMSCs at different flow velocities. A negative relation was found between the flow velocity and the cell-capture efficiency. CE denotes capture efficiency.

The flowing MagMSCs were substantially attracted to the site where the magnetic pole was positioned. Control experiments performed in the absence of an applied magnetic field demonstrated minimal accumulation (data not shown). The capture efficiency (CE) was 85.0% ± 3.6%, 68.7% ± 4.7%, 11.3% ± 4.5%, and 1.6% ± 0.75% when the flow velocity was 4, 20, 100, and 500 mm/s, respectively (Figure 4). The accumulation of cells decreased as the average velocity ($\bar{v}$) increased (*r* = −0.770; *P* = 0.003), indicating that MagMSCs can be significantly magnetically attracted into a low-flow site, such as the venous or microcircular system, but can hardly be concentrated under fast-flowing rates such as those of the arterial system.

### Perfusion area of dye injection via the transjugular cardiac vein in AMI rats

**Figure 5:**
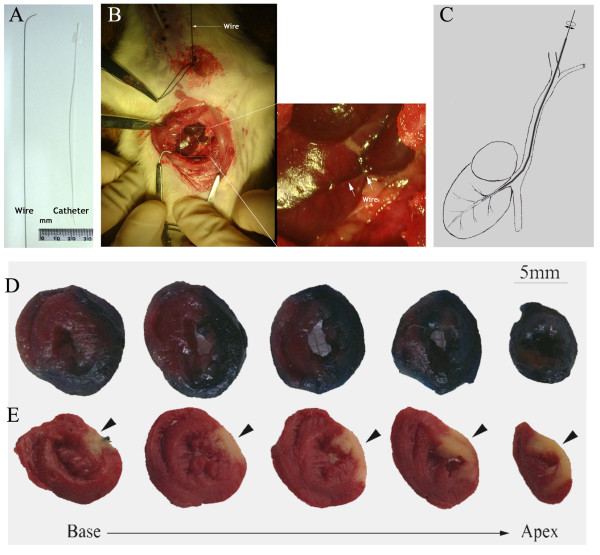
**Transjugular cardiac vein retroinfusion and its perfusion area in LAD-ligated hearts.** After the ligation of the left anterior descending coronary artery, a wire with a properly bent end **(A)** and a tube were advanced to the left cardiac vein **(B, C)**. The retrograde-infused Evans blue dye was distributed mainly at the left ventricular free wall **(D)**, covering the infarcted anterior wall, as evidenced by a staining defect in triphenyltetrazolium chloride staining **(E)**. Arrowhead denotes the anterior wall of the left ventricle.

On performance of the transjugular cardiac vein retroinfusion technique (Figure 5A-C), the retroinjected Evans blue solution was clearly extended throughout the layers of the left ventricular free wall. In contrast, the interventricular septum and the right ventricular free wall were unstained (Figure 5D). Although the perfusion area via the left cardiac vein is larger than the infarct area introduced by LAD occlusion, the perfusion area covered the infarct area of the anterior wall (Figure 5E).

### Magnetically enhanced MagMSC retention in the rat heart

**Figure 6:**
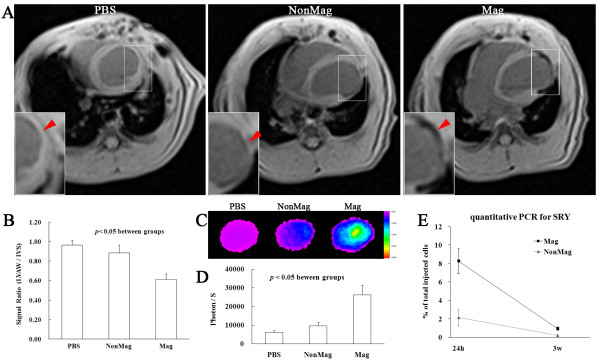
**Magnetically enhanced MagMSC retention in the rat heart. (A, B)***In vivo* MRI T_2_ fl2d cardiac images at 24 hours after injection. The anterior LV walls of cell-treated animals showed relative hypointensity that was especially obvious in the Mag group. **(C, D)***In vitro* fluorescence images of extracted heart at 24 hours after injection. Significantly escalated fluorescence signals were observed in the Mag group compared with the NonMag group. The PBS group exhibited background signal and was used as a control. **(E)** Quantitative PCR of the male-specific *SRY* gene at 24 hours and 3 weeks after cell infusion.

Twenty-four hours after retro-transplantation with or without magnet targeting, i*n vivo* cardiac MR imaging was performed to examine the transplanted cells. Because of the limited number of cells infused, no signal void was detected in all animals on T_2_ fl2d contrast-enhanced images. However, relative hypointensities representing labeled MSCs were observed at the anterior LV walls in cell-treated groups (Figure 6A). Semiquantitative analysis showed that the relative signal intensities of the anterior wall in the Mag group were lower than those of the NonMag group (0.61 ± 0.06 versus 0.88 ± 0.08%; *P* < 0.01) (Figure 6B).

After cardiac MR imaging, i*n vitro* fluorescence imaging of extracted hearts was performed. The fluorescence signals in the Mag group were approximately 2.73 times higher than those of the NonMag group (26,222 ± 5,102 versus 9,620 ± 1,930 photon/S; *P* < 0.001), whereas the signal was 6,155 ± 953 photon/S in the PBS group, which was used as a negative control (Figure 6C). These results reflected that the application of the NdFeB magnet-enhanced cell retention at the target site.

To avoid nonspecific imaging of the cells stained with iron and DiR, quantitative PCR for the male-specific *SRY* gene was performed at both 24 hours and 3 weeks after cell infusion. The Mag group exhibited a 2.87-fold greater cell-retention rate than the NonMag group at 24 hours after cell infusion (*P* < 0.001). Three weeks later, both groups experienced a substantial decrease in surviving cells. However, the Mag group still exhibited enhanced cell engraftment relative to the NonMag group (*P* < 0.001) (Figure 6D).

### Distribution of MagMSCs within the rat heart

**Figure 7:**
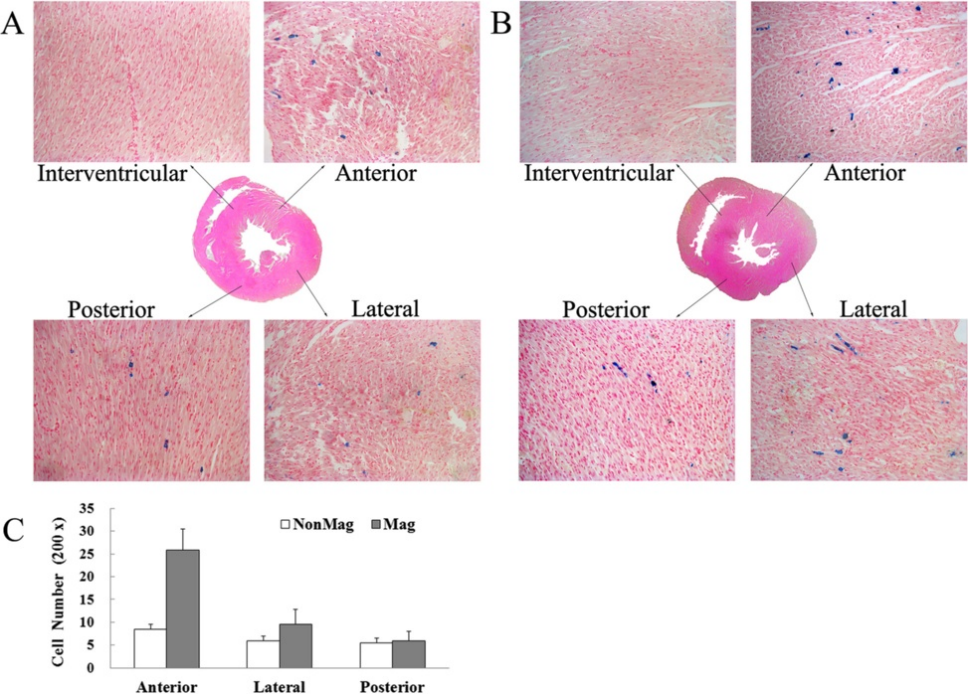
**Prussian blue staining for iron-positive cells at 72 hours after injection.** More transplanted cells (blue) were observed in the left ventricular anterior wall in the Mag group than in that of the NonMag group. More transplanted cells resided in the anterior wall than in both the lateral and the left ventricular inferior walls in each group. **(A** to **B)** Representative Prussian blue staining of ventricle from the NonMag group **(A)** and the Mag group **(B)**. **(C)** Quantitative analysis of transplanted cells.

Further to determine the distribution of MSCs in the myocardium, extracted hearts were further subjected to Prussian blue staining 72 hours after cell injection. In either group, more cells containing Prussian blue–positive particles were distributed in the anterior wall than in both the lateral and the inferior walls of the left ventricle, whereas no blue-stained cells were detected in the interventricular septum and the right ventricular free wall. It was not surprising that in the Mag group, the numbers of transplanted cells in the anterior wall were approximately 3.04 times higher than those of the NonMag group (25.8 ± 4.7 versus 8.5 ± 1.98; *P* < 0.001). However, no significant increase was noted in the numbers of transplanted cells in either the lateral or the inferior wall (Figure 7). The data suggest that the application of a magnetic field effectively directed the retrograde cell transplantation into the heart, especially to the ischemic target site, when the magnet was placed on the surface of the infarct area.

### Morphometric and functional changes

**Figure 8:**
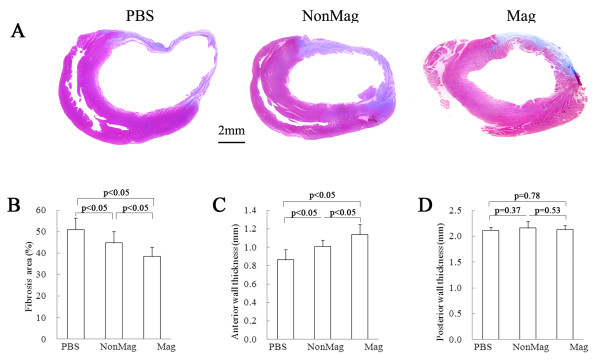
**Morphometric analysis of LV at 3 weeks after injection. (A)** Representative Masson trichrome–stained myocardial sections from each group. Scar tissue and viable myocardium are identified in blue and red, respectively. **(B to D)** Quantitative analysis of LV morphometric parameters, including risk area, anterior (infarcted) wall thickness, and posterior (noninfarcted) wall thickness. The Mag group had a smaller risk area and thicker infarcted walls than did the NonMag group or the PBS group.

Compared with the PBS group, the two cell-treated groups exhibited better heart morphology. The protective effect was greatest in the Mag group, which had a smaller risk area and thicker infarcted walls than the NonMag group (Figure 8).

**Figure 9:**
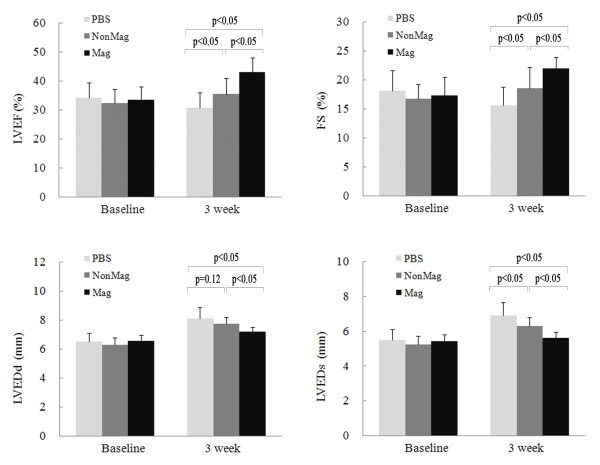
**Echocardiographic assessment of LV at 3 weeks after injection.** LVEF, left ventricular ejection fraction; LVFS, left ventricular fractional shortening; LVEDd, left ventricular end diastolic dimension; LVEDs, left ventricular end systolic dimension.

LVEF at baseline did not differ among the three groups, indicating a comparable degree of initial injury (data not shown). At 3 weeks after surgery, except for no difference in LVEDd between the Mag group and the PBS group (*P* = 0.02), the differences among the three groups reached statistical significance (*P* < 0.05) in all echocardiographic parameters including LVEF, LVFS, LVEDd and LVEDs (Figure 9). These results indicated that magnetically enhanced MagMSC retention translated into additional functional benefits for retrograde cellular therapy in the rat model of AMI.

### Angiogenesis

**Figure 10:**
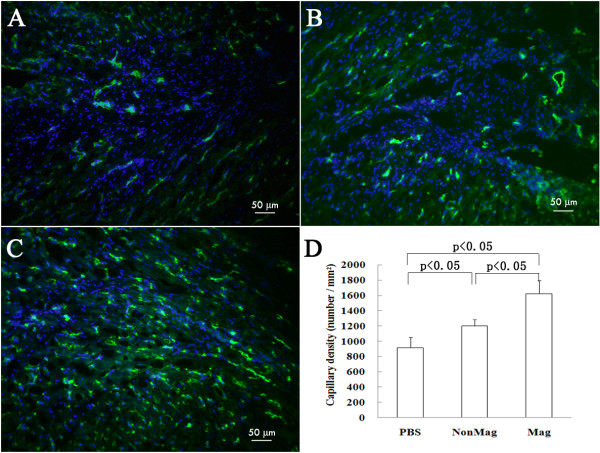
**Angiogenesis at 3 weeks after injection. (A to C)** Representative CD_31_-immunostained blood vessel (green) in the periinfarct region from the PBS **(A)**, the NonMag **(B)** and the Mag groups **(C)**. Blue indicates the DAPI nuclear stain. **(D)** Quantitative analysis of blood-vessel density in the periinfarct region.

To determine the mechanisms underlying the beneficial effects of cell transplantation, we investigated the effects of cell transplantation on neovascularization in the infarcted hearts. At 3 weeks after treatment, blood-vessel density (number of capillaries per mm^2^) in the periinfarct region was based on CD_31_ immunostaining and was highest in the Mag group (1,558 ± 166; *P* < 0.05) compared with the PBS group (917 ± 137) and the NonMag group (1,200 ± 85) (Figure 10). However, few cells stained positive for both the endothelial cell marker CD31 and the cardiomyocyte markers TnT and DiR (data not shown). These data suggest that transplanted MSCs may promote angiogenesis through a paracrine effect, contributing to the observed beneficial effects associated with cellular therapy.

## Discussion

Cellular therapy via retrograde coronary infusion, with the ability to safely deliver large numbers of cells regardless of occluded or diffusely stenotic coronary arteries, offers an attractive option for the treatment of heart diseases. Its feasibility and safety have been demonstrated clinically and experimentally [1-6]. However, the efficiency of retrograde delivery into the heart was considered the lowest when compared with antegrade coronary and intramyocardial injection-based delivery techniques [7-9]. Poor cell retention has been the main hurdle in establishing retrograde coronary infusion as the preferred route for cell delivery. Previous data suggest that poor retention is caused mainly by the rapid loss of a high proportion of cells into the systemic circulation (washing-out effect) within a few minutes [29,30]. Here, we demonstrated that brief (10 minutes) magnetic attraction successfully attenuates cell loss during injection. The increased cell retention is confirmed by the combination of quantification of the injected cells, Y chromosome-specific PCR, and MRI cell imaging. The use of the SRY marker prevented the mistaken conclusion that the grafted cells were still present, whereas iron released on cell death has been engulfed in macrophages and has subsequently given rise to false-positive signals [16]. Notably, this transient magnetic targeting seemed to have a beneficial effect on subsequent cell-therapy outcomes, and both cell engraftment and functional benefits were enhanced. Even though the magnetic targeting boosts cell engraftment at 24 hours and 3 weeks, the absolute number is still very low. Therefore, we speculated that indirect regeneration mechanisms (for example, paracrine effects) dominate the observed functional benefits.

The present study is the first to introduce magnetically targeted cellular cardiomyoplasty to the retrograde coronary-delivery strategy. Previous investigations of magnetically targeted cell delivery for cardiovascular applications are limited to intramyocardial and antegrade intracoronary routes [15-17]. Magnetic attraction can attenuate cell loss via venous drainage, increase cells’ intravascular stay, and enhance the formation of multicellular clusters, which was considered the theoretic basis for cellular magnetic targeting [15]. However, the magnetically guided cell-mass formation may increase the risk of coronary embolism after antegrade delivery [31], especially when relatively large cells such as MSCs (approximately 20 to 25 μm in diameter) are used. The more cells accumulated, the greater the risk of embolism became. On the contrary, retrograde intracoronary delivery could avoid this complication while allowing safer access to ischemic myocardium. In addition, our *in vitro* experiment demonstrated that the number of magnetically accumulated cells was inversely proportional to the flow velocity. Higher cell retention may be reasonably expected from the retrograde venous route than from the antegrade arterial route. Therefore, we speculated that retrograde coronary administration rather than antegrade coronary infusion may serve as an optimal candidate for cellular magnetic targeting. However, further studies are required to verify our reasoning.

Because of the inherent attenuation of the magnetic flux density [10], a magnet should be positioned near the target site to maximize the targeting effect. During the conventional retrograde coronary delivery in small animals, the direct insertion of a coronary sinus is needed [4,32]. The counterclockwise rotation of the heart made it technically difficult to put an external magnet simultaneously on the infarcted anterior wall in a rat. In this study, we adopted a new transjugular cardiac vein retroinfusion technique that we recently developed in which cells could be injected via a fine tube inserted into the left jugular vein [18]. In this way, inversion of the rat heart was no longer needed, and the magnet could therefore be easily positioned on the target site of the infarcted anterior wall.

An inconsistency in territory occurred between the arterial branches and the venous branches [33], and the territory of the left cardiac vein is not identical to that of the LAD. However, the perfusion via the left cardiac vein can cover the infarcted myocardium, as shown in this study; this is considered to be the theoretic basis of retrograde cellular therapy for LAD-related AMI [4]. Moreover, the infarcted tissue lacks blood flow and mechanical contraction, which decreases the loss of transplanted cells by means of washing out and squeezing out [34]. This finding could explain the relative large numbers of cells transplanted in the left ventricular anterior wall in our study, even without an external magnet. Based on this observation, an external magnet with a surface area similar to the infarct zone that was superpositioned to the injured tissue further enhanced the cell retention in the infarcted anterior wall.

The data concerning the optimal dosage for retrograde cellular therapy are limited, especially in small animals such as rats. The reported retrograde dosages of skeletal muscle precursor cells rather than MSCs varied as widely as 1 × 10^6^[4,32], 5 × 10^6^[1], and 2 × 10^6^ cells/100 g body weight [35]. The dosage of MSCs used in our study was 1 × 10^6^, which was the usual dosage used in antegrade coronary delivery in rats and was identical to the number of skeletal muscle precursor cells reported by Suzuki [4,32]. However, relatively large numbers of cells may be acceptable because retrograde intracoronary delivery bears minimal risk of coronary embolism. Although the magnetic technique could reduce cell dosage once the optimal dosage for therapeutic cell therapy is agreed in a specific model, further studies aimed at clarifying the cell dose-effect relation and identifying the optimal dose for retrograde cell infusion with or without magnetic targeting are warranted.

Some limitations of the present study exist. First, we did not explore the detailed relation between different magnetic intensities and targeting efficacy; further studies are warranted to identify the optimal magnetic intensity and to determine the targeting duration at which the optimal retention and distribution of donor cells is achieved. Second, because the retrograde coronary delivery commonly adopts a nonsurgical catheter-based technique in human beings [5], a spatially focused magnetic targeting strategy recently proposed by us [36,37] will be required for the translation of our results to a clinical setting. Third, this study failed to clarify the kinetics of cell extravasation in different time after cell infusion. Until now, the data concerning MSCs extravasation with venous delivery are limited and should be explored in future studies. Finally, because of the inability of the fluorescence devices available for performing *in vivo* imaging in rats, cell-retention dynamics were not analyzed to minimize the deaths of the animals.

## Conclusion

This study highlights that the cell retention and subsequent functional benefits via retrograde coronary infusion can be significantly enhanced by magnetic targeting in a rat model of myocardial infarction. Additional bench work is warranted to optimize targeting parameters and to assess the relevance of this approach in a clinically relevant large-animal model.

## Abbreviations

AMI: Acute myocardial infarction; B: magnetic flux density; CE: capture efficiency; Fr: magnetic attraction force on MagMSCs in the r direction; LAD: left anterior descending coronary artery; LVEDd: left ventricular end-diastolic dimension; LVEDs: left ventricular end-systolic dimension; LVEF: left ventricular ejection fraction; LVFS: left ventricular fractional shortening; MagMSCs: magnetic SPIO-labeled MSCs; MRI: magnetic resonance imaging; MSC: mesenchymal stem cell; NdFeB: Neodymium-iron-boron; SPIO: superparamagnetic iron oxide nanoparticles; SRY: region Y gene; TTC: triphenyltetrazolium chloride.

## Competing interests

The authors declare that they have no competing interests.

## Authors’ contributions

ZH conceived of the study, participated in its design, and drafted the manuscript. YS carried out all cell culture, performed surgical procedures, and was involved in drafting the manuscript. AS and NP analyzed the distribution of the magnetic flux density, and performed *in vitro* magnetic study. GH participated in PCR experiments.HZ and BH participated in surgical procedures and animal experiments. JX performed the optical imaging. YS and JM performed the magnetic resonance imaging. XY performed all echocardiographs. YZ was involved in revising the manuscript. JQ and JG conceived of the study, participated in its design and coordination, and critically revised the manuscript. The first three authors contributed equally to this work. All authors read and approved the final manuscript for publication.
